# The ecological momentary assessment approach and the use of big data to analyse possible effects of urbanisation on mental health

**DOI:** 10.1192/j.eurpsy.2021.49

**Published:** 2021-08-13

**Authors:** G. Menculini, I. Pigliautile, P. Moretti, F. Cotana, A.L. Pisello, A. Tortorella

**Affiliations:** 1 Department Of Psychiatry, University of Perugia, Perugia, Italy; 2 Department Of Engineering And Ciriaf Interuniversity Research Centre On Pollution And Environment Mauro Felli, University of Perugia, Perugia, Italy

**Keywords:** bipolar disorder, environment, Big Data, urbanisation

## Abstract

**Introduction:**

Smart healthcare monitoring allows detecting health conditions using Big Data, namely aggregated data concerning physiological and behavioral parameters. The continuous collection of data from smart-devices performed by the Ecological Momentary Assessment approach represents a promising application of Big Data.

**Objectives:**

This preliminary study was aimed at developing a research protocol focused on the use of Big Data in evaluating the impact of urban environment, affected by a variety of potentially damaging anthropogenic actions, on illness relapses in Bipolar Disorders (BD).

**Methods:**

This pilot study was designed by researchers from Departments of Psychiatry and Engineering (CIRIAF), University of Perugia. Environmental, physiological, and behavioral parameters and smart-devices aimed at collecting Big Data were identified. Subjects aged 18-65, affected by BD in current euthymic state referring to the University/General Hospital of Perugia will be recruited.

**Results:**

Subjects will undergo a baseline visit and three monitoring visits during one year. Wearable devices will be provided for collecting data about environmental and physiological parameters. Behavioral data will be collected through smartphone accelerometers, GPS, and overall smartphone use. Big data will be stored into an online platform that will provide real-time feedback concerning the recorded variables. Clinical information concerning BD relapses will be collected. Machine learning techniques, integrated to deterministic analysis of urban environmental conditions, will be used to create possible predictive models for BD relapses.

**Conclusions:**

The present project could allow the creation of a new operative platform for a better health management system correlating real-time Big Data to specific clinical features of BD.

**Disclosure:**

No significant relationships.

